# Hippocratic concepts of acute and urgent respiratory diseases still relevant to contemporary medical thinking and practice: a scoping review

**DOI:** 10.1186/s12890-020-01193-9

**Published:** 2020-06-10

**Authors:** Georgios Stefanakis, Vasileia Nyktari, Alexandra Papaioannou, Helen Askitopoulou

**Affiliations:** 1 Faculty of Medicine, University of Crete, Heraklion, Crete Greece; 2 Department of Anaesthesiology, University Hospital of Heraklion, Diastavrosi Vouton-Stavrakion, Voutes, 71110 Heraklion, Crete Greece

**Keywords:** Acute respiratory diseases and emergencies, Airway obstruction, Empyema, Hippocrates, Pleurisy, Pneumonia

## Abstract

**Background:**

The collected works of Hippocrates were searched for concepts on the diagnosis, prognosis, and treatment of acute and urgent respiratory diseases, with the objective to trace their origins in the *Hippocratic Collection*.

**Methods:**

A scoping review was performed to map out key concepts of acute and severe respiratory diseases in the entire *Hippocratic Collection.* The digital library *Thesaurus Lingua Graeca (TLG)* was researched for references in the entire *Hippocratic Collection* regarding the epidemiology, pathophysiology, prognosis, diagnosis and treatment of acute respiratory diseases; then, the relevant texts were studied in their English translation by the Loeb Classical Library.

**Results:**

Hippocratic physicians followed principles of treatment for pneumonia and pleurisy, still relevant, such as hydration, expectoration, analgesia and prompt mobilisation. Other approaches, including the inhalation of *“vapours through tubes”* in angina, can be considered as forerunners of modern medical practice*.* Thoracic empyema was diagnosed by shaking the patient and direct chest auscultation after *“applying your ear to his sides”*. In case of an emergency from upper airway obstruction, urgent insertion of primitive airway equipment, such as a small pharyngeal tube, was applied.

**Conclusions:**

The main Hippocratic concepts on four still common acute and urgent respiratory diseases −pneumonia, pleurisy, thoracic empyema and upper airway obstruction− were identified and most of them were found to be in agreement with contemporary medical thinking and practice.

## Background

The Greek physician Hippocrates lived during the fifth century before common era (BCE), the golden age of the Greek civilisation, a period to which the birth of Greek rationalism and the *“extraordinary awakening of intellectual interest”* for both science and philosophy are attributed [1, 2]. Hippocrates, a contemporary of many *“enlightened minds”*, such as Socrates, Plato, Pericles, Sophocles, Thucydides and Euripides, is widely regarded as the *“Father of Medicine”*, as he was the first person to separate medicine from religion, philosophy and superstition and to show the way towards clinical observation, rational reasoning and interpretation of accumulated data [2, 3, 4a, 5]. He clearly explained for the first time that diseases had natural rather than supernatural causes [6]. Diseases were given a logical interpretation and were no longer considered to be a punishment from divine wrath [5].

Several of his writings introduced ethical and moral values into medicine still inspiring for current medical practice [7, 8]. According to Fielding H. Garrison: *“The eminence of Hippocrates is three-fold: he dissociated medicine from theurgy and philosophy, crystallized the loose knowledge of the Coan and Cnidian Schools into systematic science, and gave physicians the highest moral inspiration they have”* [9a].

Hippocratic medicine was transmitted through the centuries by the collection of about 60 manuscripts, known as the *“Hippocratic Collection”* or *“Corpus Hippocraticum”*. Although they share a common spirit of rationalism, the greater part of the manuscripts differ in glossology, date of composition and in the views they advance, except that all are written in the Ionic dialect, *“the standard literary language of philosophy, medicine and science at the time”* [4b, 8, 10a]. This heterogeneous character indicates, most certainly, that not all of them were written by Hippocrates himself. Actually, only a few of them are generally attributed to Hippocrates’ genius [10b]. Taking into account both the fact that there are texts representative of the medical school of Cos, to which Hippocrates belonged, and the disparate nature of the *Hippocratic Collection*, Jacques Jouanna speaks of *“a collection of writings both heterogeneous and homogeneous”* [10a].

The Hippocratic writers classified diseases as acute and chronic [9b]. They were mainly concerned with acute conditions, because *“acute diseases cause many times more deaths than all others put together”* [11a]. The treatise *Regimen in Acute Diseases* has specified the *“acute”* diseases as follows: “*Now the acute diseases are those to which the ancients have given the names of pleurisy, pneumonia, phrenitis, and ardent fever”* [11a]. Despite clear descriptions of emergency cases in the *Hippocratic Collection*, it is interesting that the word “emergency”, *“epeigon”* in Greek, is never mentioned in its treatises. The writer of the treatise *Regimen in Acute Diseases* also admires the qualities of the physician who can manage acute diseases, as all physicians are not efficient to the same extent in dealing with them: *“I should most commend a physician who in acute diseases, which kill the great majority of patients, shows some superiority”* [11a].

In an attempt to explain the causes of diseases, the Hippocratic treatises often adopted the *“humoural doctrine”*, which is the balanced mixture of four main body humours –blood, phlegm, yellow bile, black bile– responsible for health, or, in case of disequilibrium, for the disease [4c]. This theory was widely accepted for centuries until it was proved to be a fallacy.

Although Hippocrates’ awareness of acute diseases is generally acclaimed, his writings have not been acknowledged as the origin of acute respiratory medicine. The aim of the present scoping review was to clarify conceptual boundaries of the diagnosis, prognosis, and treatment of acute and urgent respiratory diseases in the collected works of Hippocrates and to test the hypothesis whether these concepts, although first described in the classical Greek era, are still relevant to contemporary medical thinking and practice [12, 13]. These concepts have not been previously comprehensively reviewed or clarified.

## Methods

A scoping review was performed to map out key concepts of acute and severe respiratory diseases in the entire *Hippocratic Collection* based on the following inclusion criteria: (a) respiratory diseases included in the definition of “acute diseases” by the Hippocratic writers, (b) respiratory diseases requiring an urgent intervention. Other respiratory diseases not meeting these criteria, such as consumption −contemporary tuberculosis− were excluded from the study.

The comprehensive digital library TLG*,* a search engine of the University of California, was used for the identification of relevant citations in the *Hippocratic Collection*. The TLG has systematically collected and digitised most literary texts written in Greek, from Homer to the fall of Byzantium, and thus has become an indispensable tool for the study of Greek literature. In a second stage all relevant references were further studied both in the original Greek text and in the classical English translation of the *Loeb Classical Library*, one of the most valid collections of classical masterpieces.

Key words used for our research included terms related to acute respiratory disesases, such as “acute diseases”, “pneumonia” and “pleurisy”, and words referring to the organs lying within the chest or neck, such as “lungs”, “bronchi”, “throat” and “uvula”.

## Results

In two treatises of the *Hippocratic Collection* the definition of “acute diseases” included two respiratory diseases: “pneumonia” and “pleurisy” [11a, 14a]. Further research identified citations of these two diseases focusing on their progress to “thoracic empyema”, a condition also requiring an urgent intervention [15a, 16]. Additional analysis of the “empyema” cases identified citations related to “angina”, *“kynanche”* in Greek, an inflammation of the throat. Some of the “kynanche” references include descriptions of airway obstruction, a true respiratory emergency [17, 18]. However, in the *Hippocratic Collection* “kynanche” was not classified as an acute condition. Finally, the search based on the key word “uvula” revealed a description of suffocation due to staphylitis [19a]. The mapping of pneumonia, pleurisy, thoracic empyema and airway obstruction in the Hippocratic treatises is presented in Table 1.

**Table 1:** Mapping of acute respiratory diseases and emergencies in the *Hippocratic Collection*

Disease	Treatises
pneumonia	Affections, Airs Waters Places, Ancient Medicine, Aphorisms, Coan Prenotions, Critical Days, Diseases I, Diseases II, Diseases III, Diseases IV, Epidemics 4, Epidemics 5, Epidemics 6, Epidemics 7, Humours, Internal Affections, Nature of Women, Places in Man, Prognostic, Regimen III, Regimen in Acute Diseases, Regimen in Acute Diseases (Appendix)
pleurisy	Affections, Airs Waters Places, Aphorisms, Coan Prenotions, Critical Days, Diseases I, Diseases II, Diseases III, Epidemics 5, Epidemics 6, Humours, Internal Affections, Letters, Places in Man, Regimen in Acute Diseases, Regimen in Acute Diseases (Appendix)
thoracic empyema	Affections, Airs Waters Places, Aphorisms, Coan Prenotions, Diseases I, Diseases II, Diseases III, Epidemics 5, Epidemics 7, Instruments of Reduction, Internal Affections, Nature of Bones, On Joints, Places in Man, Prognostic, Prorhettic II, Regimen in Acute Diseases, Regimen in Acute Diseases (Appendix)
airway obstruction	Diseases II, Diseases III, Regimen in Acute Diseases (Appendix)

### Pneumonia

In the Hippocratic treatises the prevalence of pneumonia was related to geography, climate and season with most people affected during winter [14a, 20]. Hippocratic physicians attributed pain and productive cough to the collection of bile and phlegm into the lungs [14b, 21a]. On the contrary, pneumonia without expectoration was thought to be due to *“dryness and both heat … and cold”* preventing the outward movement of bile or phlegm [15b].

Pneumonia presented with fever and cough with sputum changing from clear to bilious and finally to purulent, while *“the patient’s breathing is rapid and hot”* [14b, 21a, 22a]. Both hemithoraces, the clavicles and the throat became painful and fissures appeared on the tongue [21a, 22a]. However, the most dangerous findings were either fever with concomitant expectoration of yellow or green sputum or abrupt cessation of expectoration [21b]. Emphasis was placed on the physician’s assessment of the severity of the disease: *“You must recognise that the disease is severe, when patients begin to expectorate sputum that is thickish, and you must clean them right then”* [15c].

The principles of treatment included the enhancement of expectoration with warm gruels and vapour-baths, along with the evacuation of both thorax and abdomen by enemas or cathartics for the management of pain and fever [11b, 22b]. At the same time care was taken to keep the lung “*adequately moist”* [14b, 22b]. For additional pain relief, the physician anointed the patient with warm oil and performed phlebotomies, also taking into account “*the condition of the body, the season, and the patient’s age and colour”* [23a].

The outcome depended on a process known as *“crisis”*, which lasted for fourteen to eighteen days [14b]. If during these *“critical days”* the sputum became *“mature”* –purulent– the patient would recover [15d]. If there was no purulent sputum or if the patient could not expectorate, death was very likely [15d]. Lack of expectoration could also lead to empyema [15d]. It is noteworthy that prognosis was favoured by the formation of abscesses in the legs during the *“maturation”* of sputum [24a]. The complication of pneumonia by diarrhoea or phrenitis –an inflammation of the hypochondria– was associated with an unfavourable prognosis [25a–b].

### Pleurisy

Pleurisy was considered to be similar to pneumonia, except that it affected only one side [21a]. The pathophysiology was also related to the humoural doctrine [14c]. The disease was described as bilious or sanguineous, if the sputum contained bile or blood respectively, or as dry pleurisy in case of dehydration of the lung [21c, 22c].

The clinical findings of pleurisy included chest pain, productive cough, tachypnoea, orthopnoea, fever and shivering [19b, 22c]. Pleuritic pain could extend from the shoulder and clavicle to the suprapubic area [15e]. *“Pleurisy in the back”* was an alarming condition, as the patient would suffer in agony, become tachypnoeic with immediate but little expectoration and pass blood-stained urine [22d]. In cases of pleurisy the physician was instructed to assess *“if the patient is different in other ways from what is normal”* [23b].

The therapeutic strategies for pleurisy were almost the same as for pneumonia [22e]. Additional treatment was more case-specific, taking into account the type of pleurisy –bilious, sanguineous, dry– and the individual patient –severity of disease, expectoration [21c, 22e].

The key to prognosis was expectoration, with “*crisis”* expected within seven to fourteen days [22c]. Removal of purulent sputum was considered to be associated with recovery, since if expectoration failed, thoracic empyema was anticipated [14d, 25c]. Pneumonia was another possible complication [15f]. There were also other prognostic signs. “*If the tongue becomes rough at the beginning, recovery from the disease is difficult.”* [22f]. However, *“if this sign appears when the disease is already advanced … the patient inevitably expectorates blood”* [22f]. If hiccups and haemoptysis were present, the patient could “*succumb on the seventh day”*, but if the patient survived, an empyema formed [22c]. The treatise *Diseases I* describes the death from pleurisy as agonising: *“The patient can succeed neither in coughing them up nor in bringing them to maturity, but his bronchial tubes are filled by the phlegm and pus in them. Then, the patient’s breathing becomes stertorous, and he exhales rapidly and only from the upper part of his chest; In the end, he becomes completely blocked up, and dies”* [15g].

### Thoracic empyema

Acute respiratory diseases, if not treated in time, could result in the formation of thoracic empyema, the net result of the collection of bile and phlegm in the lung and failed expectoration [15h, 21d]. In terms of diagnosis, the emphasis was on dyspnoea, fever, pain, non-productive cough and plectrodactyly, the famous Hippocratic drumstick fingers, a term still valid [19c, 22g, 24b]. The collection of pus in the thoracic cavity was revealed by the presentation of fever and rigour or when *“a heaviness took the place of the pain”* in the chest [24c]. The presence of diarrhoea caused further clinical deterioration and death [15h].

The thorough examination of the suspected side provided evidence of empyema: *“Turn the patient... inquire whether he has a pain in the side. And if one side be somewhat hotter than the other, ask the patient, while he is lying on the sound side, if he feels a weight hanging from the upper part. Should this be so, the empyema is one-sided, on whichever side the weight occurs”* [24d]. The diagnosis was further facilitated by direct chest auscultation after shaking the patient: *“If the patient does not expectorate... set him on a steady chair; let someone else hold him by the shoulders, and you shake him, applying your ear to his sides”* [22h]. The physician searched for “*a sound in the flank as if in a wineskin”* from the pus splashing [21e]. What is even more impressive, is the high degree of clinical suspicion recommended if there were no such auscultatory findings: *“If the pus, because of its thickness, does not fluctuate or make any sound in the chest, but the patient draws his breath rapidly, his feet swell up, and a mild cough is present, do not be deceived, but know well that his chest is full of pus.”* [22i].

Treatment relied on both conservative management and invasive procedures [26]. Enhancement of expectoration and chest evacuation with gruels, warm baths, inhalation of specific vapours “*through a reed”* and avoidance of prolonged immobilisation were the goals of conservative management [19d, 21f, 22h]. Cauterisation or incision was the main invasive option. The physician was very cautious when deciding where to operate on, ensuring that the patient would not change position or cough to avoid damaging the diaphragm [22j]. Special attention was also paid to prevent the massive discharge of the pus collection [25d].

The prognosis was not easy to determine, as the outcome depended on multiple factors relating to the primary disease and the patient’s reserve [15i]. Prompt initiation of treatment was important, as delays could prove fatal [15h]. The outflow of *“pure and white”* pus, possibly with *“streaks of blood”*, increased the likelihood of recovery, whereas *“muddy and evil-smelling”* pus or *“yolk-coloured”* pus on the first day, which later became *“thick, slightly yellow-green and stinking”* was associated with impending death [19e, 25e]. Positive prognostic signs were normal breathing, the absence of pain, unhindered cough and expectoration, lack of localised temperature, as well as normal hydration status and body elimination products. On the other hand, negative predictive value was attributed to strenuous and painful breathing, difficulty in expectoration, evidence of dehydration and thoracic or abdominal inflammation with concomitant peripheral vascular shutdown, sleep disturbance and affected products of elimination. Such evidence was suggestive of death within fourteen days [24e]. The physician could also predict on which days the empyema would *“break”* leading to pus removal [24f]. Death from empyema was related to suffocation: “*The patient chokes, and has more and more difficulty breathing; his breathing is stertorous, and he exhales only from the upper part of his chest. In the end, he becomes completely blocked up by the sputum, and dies”* [15h].

### Airway obstruction

The true respiratory emergencies in the *Hippocratic Collection* are cases of airway obstruction caused by angina, *“kynanche”* in Greek [17, 18]. Such cases presented with a change in shape, softness, flexibility and colour of the tongue from *“filling”* of the sublingual vessels. This could be a description of tongue oedema, where the recommended treatment was a combination of conservative measures –drinks, lozenges, gargles, anointing the neck with wax and washing with soft sponges and warm water– and invasive ones –phlebotomy [23c].

Another equally worrying case of airway obstruction prompted more urgent action: *“With angina … the person chokes and seems to have something like an apple caught in his throat... he is unable to swallow... His eyes hurt and protrude as in those that are being strangled... His face, throat and neck are distended”* [22k]. In addition to phlebotomy and enhancement of expectoration, an urgent intervention was required to secure air entry: *“Insert tubes into the throat behind the jaws, in order that air may be drawn into the lung... have the patient draw the vapours through the tubes into his nostrils, in order to discharge the phlegm”* [22l]. This citation emphasises the importance of the airway patency achieved with the insertion of *“tubes”*, a primitive means for airway management, analogous to current nasopharyngeal airways or even tracheal tubes [27].

Staphylitis, the inflammation of the uvula, was another condition often associated with airway obstruction [19a]. Treatment relied upon surgical intervention by *“cutting away the extremity”* of the uvula, followed by gargles from *“water prepared from herbs”* and the intake of cold flour and water [19a].

## Discussion

This scoping review revealed the origins of current key concepts of acute respiratory diseases and emergencies in the *Hippocratic Collection*. In these 2500 years old treatises references to several acute and urgent respiratory diseases common until nowadays were identified and studied. The accurate clinical observation by the Hippocratic physicians of the patient’s symptoms, breathing pattern and auscultatory findings remains the cornerstone of the modern approach for the management of acute respiratory diseases and emergencies.

Hippocratic physicians did not rely for diagnosis and prognosis only on observing symptoms and signs, but also on a high degree of clinical suspicion and judgement for an accurate assessment. For example, the absence of typical auscultatory findings in cases of suspected thoracic empyema did not always exclude its presence. Moreover, the step-by-step meticulous approach of empyema by thoracocentesis was amazing. Not only could Hippocratic physicians diagnose diagnose fluid in the chest by auscultation, but they also recognised that the fluid should be allowed to flow away slowly, in order to minimise the risk of collapse. The process of the simultaneous diagnosis and treatment is a precursor of the modern concept of *“theragnostics”*, the combination of diagnostics and therapeutics, with the latter based on the former’s results [28]. According to the Hippocratic scholar WHS Jones this is another example of the “*outstanding genius, who inherited much but bequeathed much more”* [4d].

The physical examination of patients suffering from respiratory diseases did not focus only on respiratory symptoms and signs. The physician also observed the general appearance of the patient for findings beyond the respiratory system –a holistic approach indicating that a disease originating from the respiratory system could progress to a systemic inflammatory response. Hippocratic medicine has been previously associated with modern evidence-based medicine [29]. This association can further be seen in the case of acute respiratory diseases, as the physician’s responsibility was to continuously assess the patient, in order to be able to reach valid conclusions.

Most of the Hippocratic principles of treatment of acute respiratory diseases are still valid. The conservative management relied on a multimodal approach, including reinforcement of hydration, expectoration, analgesia and prompt mobilisation of the patient. Moreover, the reference to the inhalation of vapours through tubes or reeds could be regarded as a precursor of modern inhalers. When invasive procedures were deemed necessary, as in thoracic empyema, the physician did not hesitate to apply even more drastic measures, such as cauterisation or paracentesis.

The individualisation of treatment, another key concept of modern medicine, was an important principle in Hippocratic medicine. Although not clearly stated as such, this was implied in the management of pneumonia and pleurisy, when patients’ demographics and reserve, as well as specific findings, were taken into consideration.

In all of these aspects of the Hippocratic approach to respiratory diseases the origins of current clinical practice in acute respiratory medicine can be traced. What is even more remarkable is the medical thinking and philosophy underlying these concepts. The physician tries to understand the factors leading to the presentation of the disease, analyses clinical symptoms and signs, provides the appropriate treatment and focuses on prognosis. This Hippocratic approach to acute respiratory diseases is in keeping with a more general axiom stated in *Epidemics I*: *“Declare the past, diagnose the present, foretell the future; practice these acts. As to diseases, make a habit of two things – to help, or at least to do no harm. The art has three factors, the disease, the patient, the physician. The physician is the servant of the art. The patient must co-operate with the physician in combating the disease”* [30].

In the above quotation the physician’s task is described in a concise and comprehensive manner by focusing on three components of the disease: the patient’s history (*“past”*), the current clinical condition (*“present”*) and the expected outcome (*“future”*). Furthermore, the Hippocratic axiom *“to help, or at least to do no harm”* calls for the physician to act for the patient’s benefit, either actively by combatting the disease or by avoiding further damage. This is the ethical principle of beneficence adopted 2500 years before this concept was introduced in contemporary medical ethics by Beauchamp and Childress [31]. The patient is central in Hippocratic medicine, while the disease is not important by itself, but in connection to the patient. The physician is there to serve the patient and *“his art”* and not for his own justification.

While key Hippocratic concepts about acute and urgent respiratory diseases are still relevant, there is no doubt that contemporary advancements of medicine have surpassed several Hippocratic theories and precepts. The Hippocratic humoural doctrine as the cause of diseases has been rejected by current knowledge of physiology. The dismissal of the humoural doctrine has steered a change in the practice of phlebotomies, applied to counterbalance an excess of the “humour” of blood.

Current views on some aspects of diagnosis and prognosis are also different. For instance, pleurisy is no longer regarded as unilateral pneumonia, but is defined as the inflammation of the pleura. Likewise, current medical thinking has disregarded odd clinical manifestations, such as leg abscesses or diarrhoea, for the progress of repiratory diseases. Furthermore, contemporary medical practice attaches equal emphasis on the diagnosis, treatment and prognosis of diseases, whereas Hippocratic physicians were mainly interested in prognosis [32, 33].

## Conclusions

This scoping review has identified the origin of current concepts about acute and urgent respiratory diseases in the *Hippocratic Collection*. It has also highlighted that the Hippocratic clinical observation and reasoning for the assessment and management of acute respiratory diseases and emergencies was well ahead of its time. Several aspects of this approach are in agreement with contemporary medical thinking and practice, which paved the way for the evolution of acute medicine. As expected not all concepts presented in these texts are still valid, the most prominent examples being phlebotomy and the humoural doctrine. However, this is a lesser issue compared to the thoughtful and advanced for their time medical concepts, which should not diminish the impact of Hippocrates and his school on acute respiratory medicine. This is a legacy to be further investigated on other topics of acute medicine in the *Hippocratic Collection*.

## Data Availability

The datasets used and/or analysed during the current study are available from the corresponding author on reasonable request.

## Abbreviations

TLG: Thesaurus Lingua Graeca
BCE: Before Common Era
